# Global Disparities in Outcomes of Pregnant Individuals With Rheumatic Heart Disease: A Scoping Review

**DOI:** 10.1016/j.jacadv.2024.101368

**Published:** 2024-10-30

**Authors:** Jenny M. Yang, Natalie Tchakerian, Candice K. Silversides, Samuel C. Siu, Rachel F. Spitzer, Wycliffe Kosgei, Nanette Okun, Rebecca Lumsden, Rohan D’Souza, Anish Keepanasseril

**Affiliations:** a Department of Obstetrics and Gynaecology, University of Toronto, Mount Sinai Hospital, Toronto, Ontario, Canada; b Department of Obstetrics and Gynaecology, Liverpool Hospital, Sydney, New South Wales, Australia; c Department of Medicine, McMaster University, Hamilton, Ontario, Canada; d Division of Cardiology, Miles Nadal Heart Centre, Mount Sinai Hospital and Toronto General Hospital, University of Toronto, Toronto, Ontario, Canada; e Division of Cardiology, University of Western Ontario, London, Ontario, Canada; f Section of Gynaecology, Division of Endocrinology, Department of Paediatrics, Hospital for Sick Children, Toronto, Ontario, Canada; g Department of Obstetrics and Gynaecology, Moi Teaching and Referral Hospital, Eldoret, Kenya; h Maternal Fetal Medicine, Department of Obstetrics and Gynaecology, Sunnybrook Hospital, Toronto, Ontario, Canada; i Department of Medicine, Duke University School of Medicine, Durham, North Carolina, USA; j Departments of Obstetrics and Gynaecology and Health Research Methods, Evidence, and Impact, McMaster University, Hamilton, Canada; k Department of Obstetrics and Gynaecology, Jawaharlal Institute of Postgraduate Medical Education and Research, Puducherry, India

**Keywords:** cardio-obstetrics, cardiovascular diseases and pregnancy, global women’s health, global health, maternal mortality, rheumatic heart disease

## Abstract

**Background:**

Rheumatic heart disease (RHD) remains as 1 of the major contributors to indirect pregnancy-related mortality and morbidity worldwide and disproportionately affects marginalized populations.

**Objectives:**

In this scoping review, the authors sought to explore the socioeconomic, cultural, and health care access-related causes of global disparities in outcomes of pregnancy among individuals with RHD.

**Methods:**

We performed a literature search of all studies published between January 1, 1990, and January 1, 2022, that investigated causes for disparate outcomes in pregnant individuals with RHD.

**Results:**

Of the 3,544 articles identified, 16 were included in the final analysis. The key reasons for disparate outcomes included lack of secondary antibiotic RHD prophylaxis; late and more severe RHD diagnosis, differences in management and antenatal care access; lack of expert and coordinated multidisciplinary care; suboptimal patient health education; inadequate access to RHD medication, intervention and surgery in pregnancy; and limited financial and economic resources.

**Conclusions:**

These findings illustrated using a life-course approach demonstrate opportunities for clinical and public health interventions to improve outcomes in this population.

Rheumatic heart disease (RHD) results from chronic autoimmune-mediated valvular damage following 1 or several episodes of acute rheumatic fever, typically following throat infection with group A streptococci. It disproportionately affects individuals in low- and middle-income countries (LMICs) as well as Indigenous populations within high-income countries, especially where poverty and overcrowding are widespread and access to health services is limited.^1^, ^2^, ^3^ The disparity in prevalence and consequences of RHD, such as heart failure, pulmonary edema, arrhythmias, and stroke, resulting in mortality and significant loss of disability-adjusted life years, represents a social inequality between and within nations.^4^, ^5^, ^6^

Pregnancy imposes a hemodynamic stress that may be poorly tolerated in patients with RHD and can result in maternal morbidity and mortality. Cardiac disease is 1 of the major contributors to indirect maternal mortality and morbidity, with 1 Ugandan study finding the percent attributable risk of heart disease on maternal mortality to be 88.6%.^7^ The maternal mortality rate among those with RHD was reported as high as 34% in another Senegalese study.^8^ Global disparities in cardiac outcomes among pregnant individuals with RHD are well-described,^9^ with most researchers reporting disease characteristics associated with the likelihood of adverse outcomes.^8^^,^^10^ However, the adverse events may not be completely attributed to disease-related clinical factors but also from the interplay between sociodemographic, policy, and cultural factors.^8^ This scoping review aims to identify the factors associated with the disparities in adverse cardiac outcomes among pregnant individuals with RHD, focusing on sociocultural and health system-related factors to conceptualize a framework that could help reduce the adverse outcomes.

## Methods

We conducted a scoping review of the literature, adhering to the Preferred Reporting Items for Systematic reviews and Meta-Analyses extension for Scoping Reviews reporting guidelines.^11^ Ethics approval was not deemed necessary due to the study type. An electronic search of Pubmed, Embase, Medline, CINAHL, Scopus, ISI Web of Science, Global Index Medicus (includes gray literature), including LILACS, WHOLIS, Africa Wide, African Journals Online, and Google Scholar, was conducted, limited to articles published in English or Spanish, between January 1990 and January 2022. Original research papers such as randomized controlled trials, observational studies, and qualitative and mixed methods research studies were eligible for inclusion. Conference abstracts and case reports were excluded. Systematic reviews, commentaries, editorials, and opinion pieces were excluded from the main analysis, but their reference lists were scanned for publications that were missed in the original search.

The search strategy included keywords relating to pregnancy, RHD, global health, social determinants of health and health inequity. Studies were eligible for inclusion if they included pregnant or postpartum individuals (up to 12 months following childbirth) with RHD and identified causes for global inequities in maternal cardiac outcomes, or they identified target areas and proposed strategies for preventing mortality and morbidity. Studies with patients with non-RHD maternal cardiac disease were still included if they also contained a significant proportion of patients with RHD. Studies that only described RHD prevalence, outcomes, or associations between clinical parameters (such as NYHA functional class, type of valvular lesion, and other clinical disease characteristics) with rates of adverse cardiac outcomes were excluded.

The final search results were exported into DistillerSR,^12^ duplicates were removed, and titles and abstracts were screened independently by J.Y. and N.T. Eligible full texts were similarly screened independently by both reviewers. Discrepancies and conflicts were resolved through discussion with A.K. and R.D. One reviewer (N.T.) completed data extraction for all included studies using a predetermined data extraction form, including information on the study population, context, main findings, and reasons for disparate cardiac outcomes in pregnant individuals with RHD. A second reviewer (J.Y.) cross-checked data entry for 25% of the included studies.

Extracted data were analyzed using the Arksey and O’Malley framework for scoping review methodology^13^ by manually identifying themes for the causes of disparate cardiac outcomes in pregnant individuals with RHD, and the studies sharing common themes were grouped accordingly. The findings were discussed between 4 investigators (J.Y., N.T., R.D., A.K.) and through an iterative process of consolidating and determining which among the themes best represented the factors associated with adverse cardiac outcomes in pregnant individuals with RHD, a final consensus of the themes was reached and approved by the other reviewers (Supplemental Table 1).

## Results

**Figure 1:**
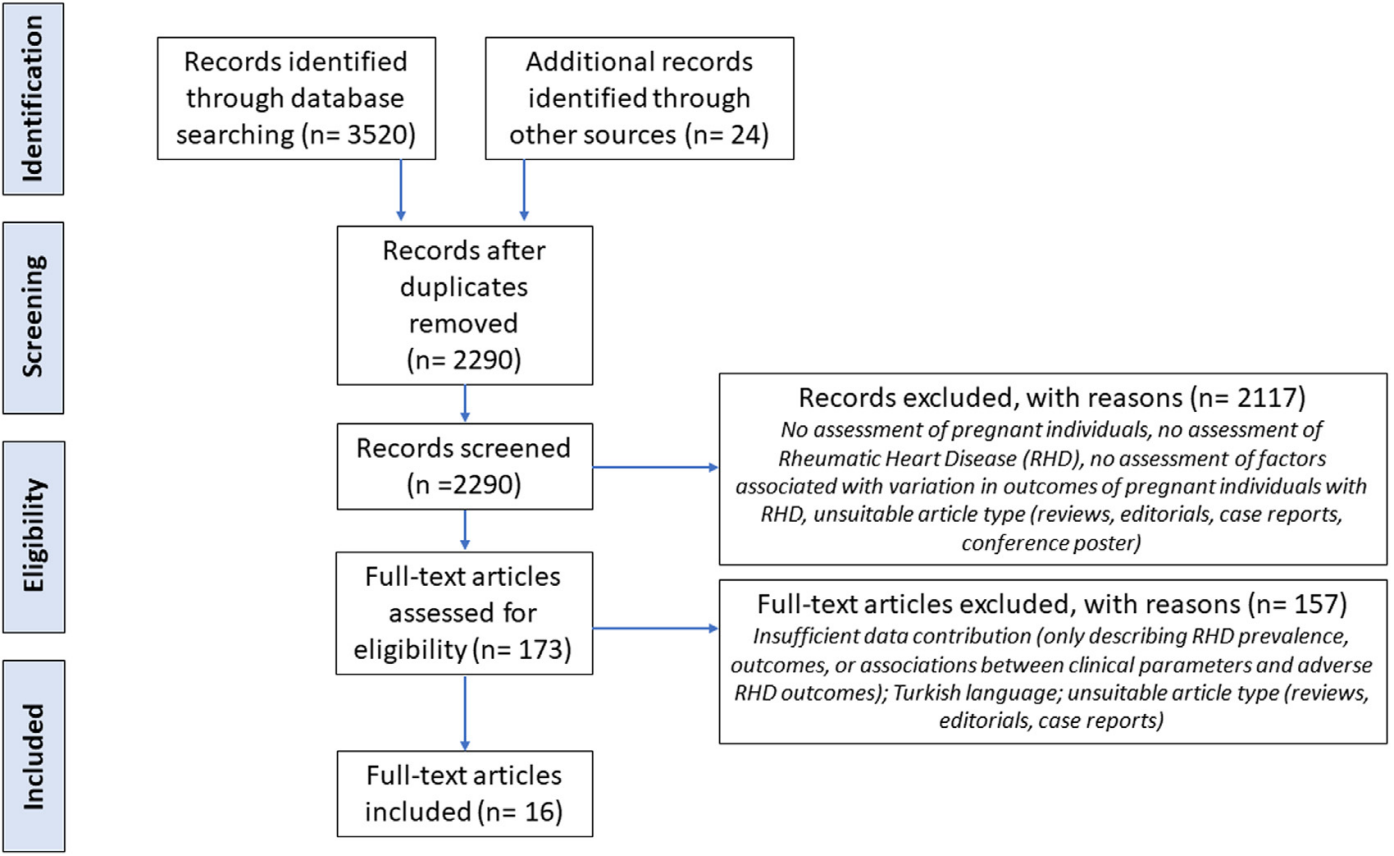
**PRISMA Flow Chart** Flow chart demonstrating selection process for the included studies using the PRISMA extension for scoping reviews guidelines. PRISMA = Preferred Reporting Items for Systematic reviews and Meta-Analyses.

A total of 3,544 articles were identified, of which 2,290 titles and abstracts were screened, 173 potentially eligible full-text articles were retrieved, and 16 articles^5^^,^^7^^,^^8^^,^^10^^,^^14^, ^15^, ^16^, ^17^, ^18^, ^19^, ^20^^,^^23^, ^24^, ^25^, ^26^, ^27^ after excluding 3 in languages other than English or Spanish, that fulfilled the review criteria were included in the final analysis (Figure 1, Table 1). They included 13 quantitative studies (7 prospective and 5 retrospective cohort studies and 1 population-based study) and 3 qualitative studies (Supplemental Table 2). The populations represented were predominantly from Africa (8 studies) and the Indigenous populations of Australia and New Zealand (5 studies). The quantitative studies included 2 multinational registries and 11 single-site studies, with a sample size ranging from 16 to 2,924 pregnant individuals. The qualitative studies were all single-site studies with a sample size from 8 to 73 participants.

**Table 1:** Summary of Included Studies

First Author, Year	Country	Context	Study Type	Sample Size	Study Objectives
Beaton, 2018	Uganda	Hospital and health centers	Prospective longitudinal cohort study	N = 3,506 screened, 58 with rheumatic heart disease (RHD)	• To determine the prevalence of pre-existing maternal heart disease using echocardiography for active case finding during antenatal care. • To determine the percent population attributable risk of cardiovascular disease to adverse maternal, fetal, and neonatal outcomes.
Belton, 2018	Australia	Primary health care settings and hospital antenatal clinics (ANCs)	Qualitative study	N = 8	• To study RHD health literacy and its impact on pregnancy, and to identify how health services could more effectively meet the needs of pregnant women with RHD.
Diao, 2011	Senegal	Tertiary hospital	Retrospective cohort study	N = 46 pregnant women with RHD	• To report maternal and fetal outcomes of pregnant women with heart disease and to identify factors associated with unfavorable maternal and fetal outcomes.
Elsayed, 2019	Egypt	Tertiary hospital	Retrospective cohort study	N = 16	• To describe the outcomes of surgical intervention in pregnant women presenting with an acute malfunctioning mechanical mitral valve.
Jamal, 2020	Mozambique	Major referral hospitals	Descriptive observational study (surveys)	N = 73	• To assess the knowledge of diagnosis and management regarding RHD in pregnant individuals among reproductive health professionals at referral centers.
Ongzalima, 2020	Australia	Tertiary center	Retrospective cohort study	N = 54	• To review the demographic, clinical characteristics, obstetric management, and major outcomes of Indigenous and non-Indigenous pregnant individuals with RHD.
Poli, 2020	Kenya	Tertiary hospital	Longitudinal cohort study	N = 91	• To identify factors related to adverse maternal and neonatal outcomes in pregnant individuals with cardiac disease.
Schoon, 1997	South Africa	Tertiary hospital	Retrospective cohort study	N = 164 pregnant women with RHD	• To describe the maternal outcome of pregnancies complicated by cardiac disease.
Sliwa, 2018	South Africa	Tertiary hospital	Prospective pilot study	N = 269 pregnant women with cardiac disease	• To investigate the effect of targeted interventions aiming at reducing peripartum heart failure admission and late maternal death in pregnant or recently postpartum individuals with pre-existing cardiovascular disease.
Soma-Pillay, 2016	South Africa	National database: confidential enquiry into maternal deaths	Retrospective cohort study	N = 169	• To determine the cardiovascular causes and comorbidities contributing to maternal mortality. • To identify avoidable factors and missed opportunities contributing to maternal mortality.
Sullivan, 2020	Australia and New Zealand (Aus/NZ)	Hospital-based maternity units	Prospective cohort study	N = 311	• To describe the epidemiology and assess the management of, and health outcomes for, RHD in pregnancy.
Van Hagen, 2018 (1)	Global	Tertiary hospitals	Prospective registry (ROPAC)	N = 2,924 (89 centers, 30 countries)	• To assess maternal and fetal outcomes of pregnancy in women with rheumatic mitral valve disease.
Van Hagen, 2018 (2)	Global	Tertiary hospitals	Prospective registry (ROPAC)	N = 2,924 (89 centers, 30 countries)	• To analyze the extent to which socioeconomic factors influence the outcome of pregnancy in individuals with RHD.
Vaughan, 2018	Australia	Maternity units in hospitals and community clinics	Population-based study	N = 192	• To identify the challenges of surveillance of pregnant individuals with RHD. • To evaluate strategies developed to enhance reporting of pregnant individuals with RHD by health services.
Vaughan, 2021	Australia	Hospitals and community-based centers	Qualitative study	N = 19	• To investigate models of care for pregnant individuals with RHD from the perspectives of health care providers, particularly identifying gaps and facilitators of optimal care.
Zühlke, 2015	Global registry (REMEDY)	Hospitals	Prospective RHD registry	N = 3,343 patients with RHD, including 73 pregnant women	• To determine patient characteristics, treatment patterns, and prevalence of adverse cardiac events of patients with RHD.

ROPAC = Registry of Pregnancy and Cardiac Disease.

**Figure 2:**
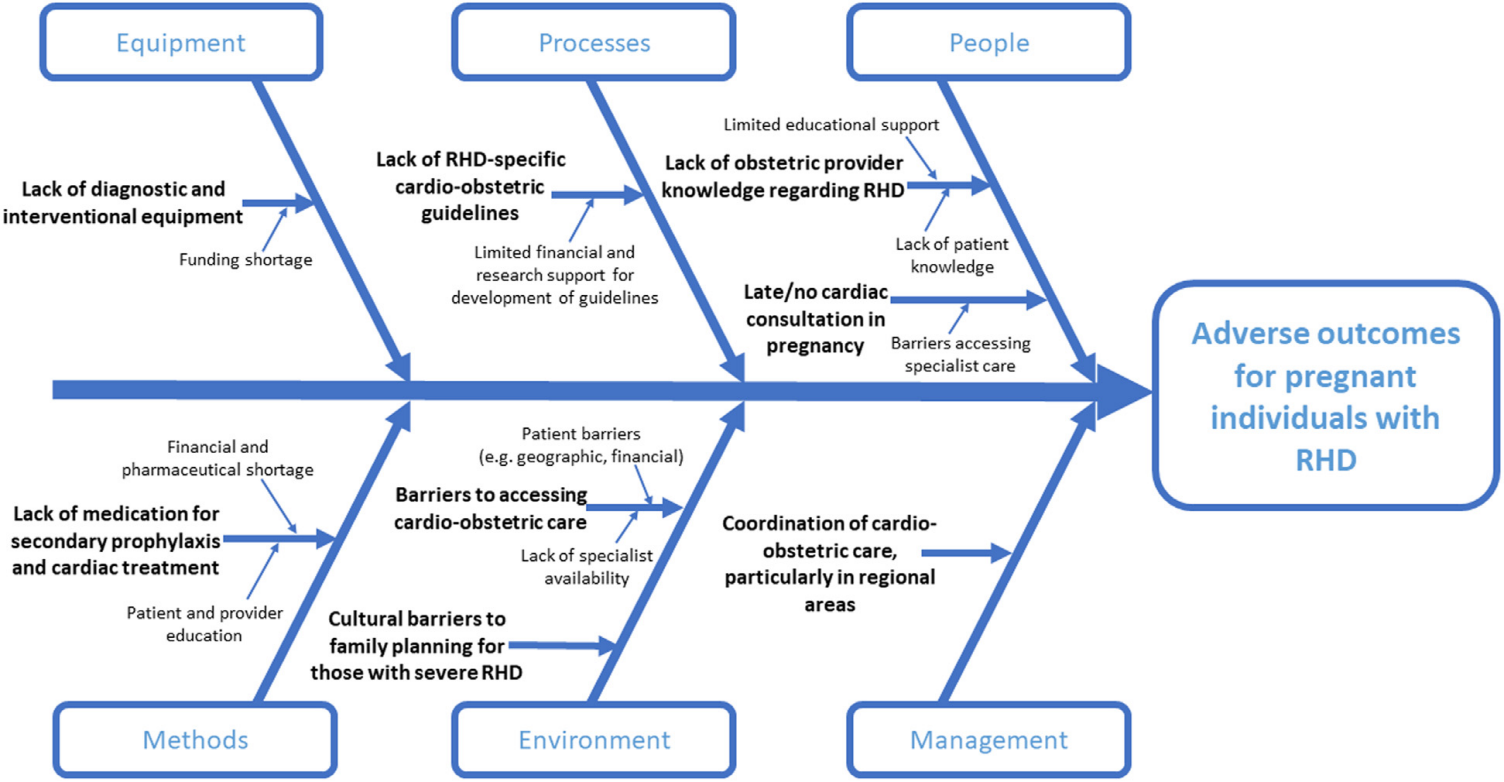
**Ishikawa Diagram for Pregnant Individuals With Rheumatic Heart Disease** Ishikawa diagram demonstrating the complex interplay of factors contributing toward adverse outcomes for pregnant individuals with RHD. MDT = multidisciplinary team; RHD = rheumatic heart disease; SDH = social determinants of health.

The reasons for disparities in cardiac outcomes among pregnant individuals with RHD identified from these studies with the potential impact and timing along the life course of the pregnant individual with RHD are presented in Figure 2, and the Central Illustration demonstrates the complex interplay between these factors.

**Central Illustration:**
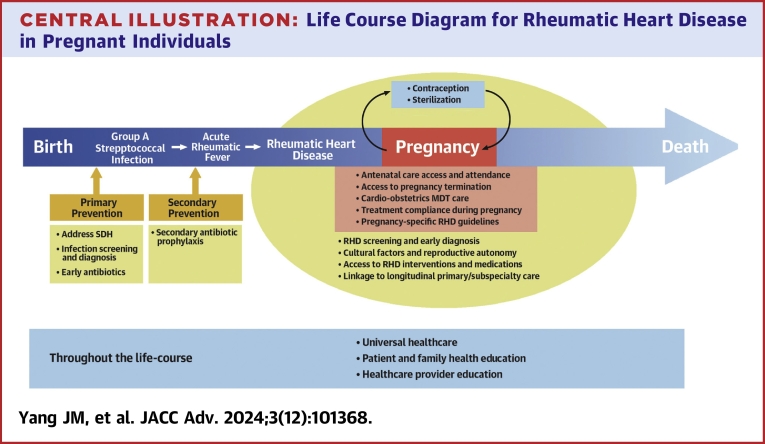
**Life Course Diagram for Rheumatic Heart Disease in Pregnant Individuals** Depiction of a life course approach to improving chronic rheumatic heart disease-related outcomes in pregnant individuals. Abbreviations as in Figure 2.

### Pre-conception and antenatal considerations

#### Secondary antibiotic prophylaxis

Secondary antibiotic prophylaxis for those with RHD, which reduces the risk of progression to severe disease, was assessed only in 1 study.^14^ Ongzalima et al^14^ found that 68.3% of Australian Indigenous and 23.1% of non-Indigenous pregnant individuals with RHD used secondary antibiotic prophylaxis either before or during pregnancy. This indicates an overall low rate of prophylaxis, with disproportionately lower rates among non-Indigenous individuals, although reasons were not explored.^14^

#### Late diagnosis of RHD

Diagnosis of RHD before pregnancy allows for cardiac interventions preconceptionally to optimize pregnancy outcomes, pregnancy planning and surveillance, and use of prophylactic therapies to prevent complications. The proportion of individuals who were diagnosed with RHD, either during pregnancy or postpartum, ranged from 13% in Indigenous Australians^15^ to 96% in a Ugandan population.^7^ Compared to individuals diagnosed before pregnancy, those diagnosed during pregnancy or postpartum reported higher rates of cardiac complications, use of cardiac medications, deterioration in NYHA functional class, and need for intensive care or coronary care unit admission, suggesting an association between late diagnosis and a more severe disease process at presentation.^7^^,^^15^ Three studies investigated health care resources in managing pregnant Indigenous Australians with RHD, noting that fragmented obstetric care and gaps in obstetric providers’ knowledge led to missed diagnoses, even among those diagnosed with RHD in previous pregnancies.^16^, ^17^, ^18^ Reasons contributing toward delayed or missed diagnoses included: 1) lack of continuity of care due to a transient or locum workforce; 2) a nonstandardized structure of echocardiography reporting; 3) absence of integrated local protocols regarding the care of pregnant individuals with RHD; and 4) noncohesive health information systems between different sites leading to loss of referrals, results, and patient records.^16^, ^17^, ^18^

#### Late engagement in obstetric care

In Australia and New Zealand, Indigenous populations with RHD had low rates of early (before 20 weeks’ gestation) attendance for antenatal care (38.5% of Australian- and 43.8% of New Zealander-Indigenous individuals). The rates of optimal antenatal care attendance, defined by 8 or more antenatal visits during pregnancy, were lower among Indigenous than non-Indigenous populations (48.7% vs 92.3%, *P* = 0.008).^14^^,^^15^ In the confidential enquiry on maternal deaths (CEMD) from South Africa, 25.3% of maternal deaths were related to RHD, and 41.5% of the maternal deaths from cardiac causes had delays in seeking care, including late engagement with antenatal care.^19^

### Clinical expertise and coordinated cardio-obstetrics care in pregnancy

#### Late or no cardiac consultation in pregnancy

Four studies, which included 625 individuals with RHD, reported on cardiac consultation during pregnancy. Despite 87% of individuals being aware of their RHD diagnosis before pregnancy, one-third did not receive any cardiac care during pregnancy and childbirth in a study among the Indigenous population from Australia and New Zealand.^15^ In another study from Kenya, less than one-half the individuals with significant cardiac disease detected during pregnancy received cardiac treatment.^20^ Lack of cardiac care was reported among two-thirds of the cardiac-related maternal deaths in the South African CEMD (2011-2013).^19^ Limited direct access to cardiology support was also highlighted as a challenge in the remote Australian context.^14^

#### Lack of multidisciplinary clinics

Integrated multispecialist clinics which provide both expertise and coordination of care by obstetricians, cardiologists, midwives, and other relevant multidisciplinary team members improve the outcomes for pregnant individuals with RHD.^21^ Such clinics will subsequently be referred to in this article using 1 of their common names, as “cardio-obstetrics clinics.” The lack of such clinics results in fragmented and poorly coordinated antenatal care by providers without knowledge of managing individuals with RHD during pregnancy and childbirth, hindering the provision of quality care in 7 studies.^14^^,^^16^^,^^18^^,^^19^^,^^21^, ^22^, ^23^ Fragmented care also contributed to a suboptimal understanding of treatment options, which hampered patient-provider rapport and trust, and affected treatment engagement and compliance for Indigenous Australians.^14^^,^^16^ In limited resource settings, the shortage of skilled experts, both high-risk obstetricians and cardiologists, was the main reason for the lack of cardio-obstetrics clinics.^14^^,^^16^^,^^18^^,^^19^^,^^21^, ^22^, ^23^

#### Provider knowledge and education regarding RHD assessment and management

A study from Mozambique demonstrated a lack of educational support and knowledge regarding the challenges during pregnancy in individuals with RHD and their care providers, which can lead to suboptimal management of pregnant with RHD.^23^ The providers of the highest level of obstetric care available in Mozambique expressed limited knowledge about managing RHD and its complications during pregnancy. Other studies also reflected this deficiency and stressed the need for resource limited context-specific guidelines for managing pregnant individuals with RHD.^14^^,^^16^, ^17^, ^18^ The South African CEMD identified the lack of expertise of medical staff, delays in referral to the appropriate level of care, and inappropriate management as avoidable factors among cardiac-related maternal deaths.^19^

### Patient health education

#### Patient health literacy and education

Diao et al,^8^ in a study from Senegal with high rates of maternal mortality among pregnant individuals with RHD, noted that most individuals were unable to recognize the risks associated with their condition and its impact on pregnancy outcomes. Low maternal educational status often leads to poor health literacy and is associated with increased maternal cardiac adverse outcomes.^8^^,^^20^ Similar observations were noted by Schoon et al,^24^ where the lack of knowledge about cardiac therapy and the challenges during pregnancy was associated with increased adverse outcomes.

#### Patient-provider communication and rapport

Another theme that emerged affecting treatment engagement and compliance was the poor quality of patient-provider interactions. A qualitative study involving health care providers reported a lack of culturally and linguistically appropriate efforts to increase health literacy regarding RHD for Indigenous Australians.^18^ Belton et al^16^ identified more specific communication issues, such as health professionals failing to use interpreters during clinical encounters when indicated, lack of vocabulary for “RHD” or “heart valves” in the Aboriginal language, and disease descriptors being poorly explained in health education materials. A lack of culturally competent care, with a resultant power imbalance between the provider and patient compounding the communication issues, resulted in patients becoming disengaged and indifferent toward health care providers.^16^ Ongzalima et al^14^ noted similar findings that a lack of understanding and trust resulted in service disengagement and nonattendance to antenatal clinics among Indigenous Australians with RHD, increasing the risk of missing diagnoses as well as complications.

### Medical treatment during pregnancy

#### Cardiac medications

Targeted medical therapies for pregnant individuals with cardiac disease, such as β-blockers during pregnancy, reduce admissions for heart failure and maternal death.^21^ However, the multinational Registry of Pregnancy and Cardiac Disease (ROPAC)^25^ reported only 40% of those with mitral stenosis (MS) and heart failure received β-blockers, the cornerstone of medical management. The reasons underlying such low rates of treatment were not elucidated.

#### Anticoagulation

Anticoagulation is recommended to prevent thromboembolic complications in individuals with rheumatic MS who have atrial fibrillation, significant left atrial dilation, a history of transient ischemic events, or in those with mechanical heart valves (MHVs). The global REMEDY registry wherein two-thirds of patients were from LMICs, demonstrated that among individuals with RHD for whom oral anticoagulation was indicated, only 20.6% of pregnant individuals and 69.5% overall were prescribed it.^26^ Of those prescribed oral anticoagulation, only 28.3% of pregnant and nonpregnant individuals were therapeutically anticoagulated.^26^ Despite having a protocol guiding warfarin use in pregnancy, poor compliance was identified in an Egyptian cohort either due to patient- or obstetrician-initiated cessation of anticoagulation.^27^ Four among 16 cases with acutely malfunctioning MHVs from thrombotic complications died in their cohort. Schoon et al^24^ identified issues with anticoagulation among those with MHV, contributing to over half the morbidities among those with cardiac disease in pregnancy in South Africa.

#### Intervention and surgery

Ideally, severe valve lesions should receive an intervention before pregnancy. For those patients who are already pregnant with severe valve disease and have symptoms refractory to medical therapy, interventions such as percutaneous balloon mitral commissurotomy (PBMC) or valve repair/replacement have been shown to optimize pregnancy outcomes.^28^ However, access to centers that offer valve interventions remains a significant challenge, especially in LMICs with limited resources.^26^ A study from Senegal on pregnant individuals with rheumatic MS reported a maternal mortality rate of 34% and highlighted the unavailability of PBMC in their institution as a likely contributory factor.^8^ In the multinational ROPAC registry, half of those with severe MS required hospital admission for heart failure during pregnancy, and only 5.8% of individuals with MS underwent PBMC during pregnancy.^25^ They also noted that even with higher rates of severe MS, valvular interventions were lower in countries with less developed economies or a lower human developmental index (HDI).^5^^,^^25^ Lower rates of cardiac interventions in LMICs compared to high- or middle-income countries were also seen in the REMEDY registry,^26^ despite more high-risk pregnant patients with indications for intervention (RHD-MS and left ventricular dysfunction) in the LMIC population.

### Financial resources

#### National

The relationship between a country’s economic status and access to cardiac valvular interventions centers with maternal cardiac complications was noted in large international registries of pregnant individuals with RHD.^5^^,^^25^^,^^26^ In the ROPAC registry, those from countries with a medium to high HDI were more likely to have heart failure, as well as worse perinatal outcomes, than those from countries with a very high HDI.^5^ The Gini coefficient, which measures economic inequality in a population, explained 4% of the variance in maternal cardiac outcomes between countries in ROPAC, reiterating the contribution of socioeconomic inequality toward the disparity in outcomes.

#### Patient/systems level

Poli et al,^20^ in a Kenyan cohort, noted that lack of health insurance contributed to poor maternal outcomes, specifically as access and affordability for admission to intensive care units in private hospitals were limited when public hospital intensive care units were full. It also contributed to restricted access to antenatal care, increasing both cardiac and obstetric adverse outcomes. Lack of advanced cardiac care facilities in remote regions and fear of birthing in a facility remote from family to receive the appropriate level of care, affected the level of care received in a study of Indigenous Australians.^14^

## Discussion

### Main findings

This scoping review focused on the nonclinical factors responsible for global disparities in cardiac outcomes among pregnant individuals with RHD. We identified 13 key factors that could inform future targets for clinical care, research, and health policy, which are adaptable to most settings, as shown in Figure 2 and Table 2 with possible strategies to target these. The influence of various characteristics along the life course highlights the importance of the periconceptional and postpartum period and directs opportunities for clinical and public health interventions to improve pregnancy and the overall health of individuals with RHD (Central Illustration). Addressing the factors identified by this review and ensuring both a life course approach and multisectoral government commitment to improve outcomes of pregnant individuals with RHD is necessary to address the insufficient progress made since the Addis Ababa communique declaration 2016, committed to RHD eradication.^6^^,^^29^

**Table 2:** Table of Interventions to Improve Outcomes of Pregnant Individuals With Rheumatic Heart Disease

Target Domain	Suggested Interventions to Improve Outcomes of Pregnant Individuals With RHD	
	Strategic Solutions	Local Initiatives∗
Preconception and antenatal considerations	• Emphasize primary prevention strategies for community prevention of RHD alongside secondary public health strategies, especially in highly endemic areas. • Increase secondary antibiotic prophylaxis by increasing population RHD screening and subsidization of treatment. • Improved RHD risk stratification scoring systems for early identification of high-risk patients to facilitate appropriate transfer to tertiary facilities and early pregnancy termination counseling.	• Increase secondary antibiotic prophylaxis by increasing health education for patients and health care providers. • Echocardiography screening for RHD *prior* to pregnancy, or as early in pregnancy period as possible.
Clinical expertise and coordinated cardio-obstetric care	• In settings without subspecialist services, ○ Establishing virtual care with cardio-obstetrics teams ○ Referral networks to tertiary care Hubs ○ Increasing capacity of generalists to care for pregnant people with RHD ○ Forming global academic partnerships to facilitate on-site specialization training	• Multidisciplinary cardio-obstetrics team management (including cardiology, maternal-fetal medicine/high-risk obstetrics, anesthesia, family practice, neonatology, nursing, midwifery, social worker, mental health support), including virtual care and capacity building. • RHD education modules for obstetric providers, including communication and cultural sensitivity training to provide health education to minoritized communities.
Patient and community health education	• Peer support groups for RHD patients and communities, with a health educational component on risks of current and future pregnancy, family planning options, and importance of RHD treatment compliance.	• Use of social and local media, cultural/religious/social channels, interpreters and/or cultural liaison officers where relevant, to educate and disseminate knowledge.
Access to medical therapies during pregnancy	• Government commitment to increase funding for training in cardio-obstetric services: skilled providers, cardiac intervention, and resources. • Universal access to family planning services, including abortion.	• Regional government subsidization and establishment of national supply chains of cardiac medications, intervention, and surgery.
Financial resources	• Universal health insurance for all, especially all individuals with RHD	• Government-supported establishment of family planning services, particularly in regional areas.

RHD = rheumatic heart disease.

∗ Local initiatives denote starting point, as they can be integrated into national health policies.

### Life-course approach to RHD management

Improving the outcomes of RHD during pregnancy requires a life-course approach to effectively reduce the burden of this disease during pregnancy and across the individual's lifespan (as depicted in the Central Illustration). The life-course approach for RHD presented by Vaughan et al^18^ in the context of the Australian Indigenous population applies to all low-resource settings. Initiatives such as screening children for rheumatic fever or heart disease through school health initiatives and antenatal screening with artificial intelligence-assisted technologies may aid in the early diagnosis and initiation of secondary penicillin prophylaxis to prevent the progression of the disease.

In addition to periconceptional, antepartum, and postpartum care, there is a need to ensure contraception access for individuals with severe RHD to avoid repeated or unplanned pregnancy. Only 3.6% of reproductive-aged women were using contraception in the REMEDY registry,^26^ and only 26% of reproductive health care providers in another study from Mozambique reported access to contraception guidance for women with RHD.^23^ Access to abortion is also critical for individuals with severe cardiac disease at high risk of maternal death,^7^ although it is complicated by the variable legality of abortion.^30^ Poor retention in medical care following RHD diagnosis, at 24% in 1 Ugandan study,^31^ impedes the opportunity to provide contraception, preconception counseling, and optimization.

### Cardio-obstetric care and the need for resource-oriented RHD-specific guidance

Lack of integrated cardio-obstetric care during pregnancy in low-resource and remote settings was noted as 1 of the important factors for the occurrence of adverse cardiac outcomes in pregnant individuals with RHD. However, this is difficult to address in settings with a shortage of cardiologists, high-risk obstetricians, and maternal-fetal-medicine specialists, with limited capacity to support the training of such specialties. Global academic partnerships such as the AMPATH (Academic Model Providing Access to Healthcare) model described by Binanay et al,^32^ which has facilitated the development of “in-house” cardiology and maternal-fetal-medicine training programs in Western Kenya with the support of faculty in North American academic institutions, is a step toward ensuring expertise, and reducing global disparities of resources.

Expertise being limited to fewer tertiary centers necessitates early and ongoing risk stratification to ensure appropriate and timely referrals to optimize the outcomes. Most of the risk stratification models in pregnancy were developed in high-income settings, where congenital heart disease is the predominant cardiac diagnosis^33^ and may not have the same accuracy in LMIC settings where RHD is more common.^8^^,^^20^ The RHD-specific risk stratification DEVI model, developed and validated in an LMIC with better predictive accuracy, is a promising step toward adopting a setting- and lesion-specific strategy.^34^

Among the various cardiac medications, the use of anticoagulation remains a significant challenge in LMIC settings, with lower prescription rates, difficulties in monitoring, and lower compliance, with consequent high complication rates during pregnancy.^26^^,^^27^ Those caring for such individuals should be aware of emerging evidence of the safety of cardiac medications, interventions, and the alternative strategies that could be used effectively in pregnant individuals with RHD in low-resource settings.^35^ This suggests the need to develop guidance for managing pregnant individuals with RHD in LMIC settings, considering the available resources, logistic support, and expertise to optimize the outcomes.

### Targeting socio-cultural barriers

Among pregnant individuals with RHD, the usage of cardiac medications and interventions is often suboptimal, and severe RHD lesions are more likely among those economically disadvantaged or lacking health insurance.^5^^,^^20^^,^^25^^,^^26^ Adopting universal health care, that could ensure equitable cardio-obstetric care, may be an important strategy to tackle these factors.

Misconceptions and concerns regarding medication side effects, as well as community stigmatization regarding chronic medication use, identified as barriers to use of contraceptives and anticoagulation in a study from Uganda, highlight the need for patient education.^36^ Cultural factors and limited reproductive autonomy, such as that described by 1 individual about her husband insisting on another pregnancy for want of a son despite pregnancy posing increased risk to her life, are common in some communities.^22^ Community stigmatization of those unable to child-bear and pressure to reproduce, with fear of—or actual abandonment by male partners, is a reason that some individuals pursue pregnancy despite being medically advised against pregnancy due to the severity of RHD.^36^ These reports highlight the need to educate individuals and their families on the impact of pregnancy on RHD and optimize the cardiac condition preconceptionally, which could improve early antenatal booking and compliance with follow-up and treatment to achieve optimal pregnancy and maternal outcomes.

### Strengths and limitations

The strengths of the study includes: 1) its strategy-based focus, aiming to identify sociocultural factors that could be targeted to improve pregnancy outcomes in individuals with RHD in contrast to most of the existing literature that focuses on disease-specific risk factors, which are often the product of societal root causes of disparate RHD-related outcomes; and 2) the multidisciplinary team of authors representing high-, middle-, and low-income countries, providing various perspectives in the interpretation of data.

The review also had some limitations. First, the included studies represent several African countries and the Indigenous populations in Australia and New Zealand, but several regions are not represented including LMICs in Asia or South and Central America. The reasons for disparities in outcomes among racialized, marginalized, and immigrant populations in the high-income countries in Europe or North America were also not available to be included. This is largely due to the significant lack of research focusing specifically on nonclinical factors that influence the outcomes of pregnant individuals with RHD, which represents the lack of recognition of the importance of such factors in RHD research. This research gap also results from the under-representation of LMICs despite bearing most of the global RHD burden, due to issues such as lack of research infrastructure and funding, highlighting the need to address the broader equity issue of equal population representation in global health research.^37^

Secondly, the lack of a uniform and longer follow-up postpartum period in various studies might have led to under-recognition of factors associated with morbidity beyond the conventional 6-week postpartum period. Finally, the lack of details on reasons for low treatment rates related to prescription, access or compliance, limited the understanding of strategies to increase the treatment rates.^14^^,^^25^

## Conclusions

Outcomes of pregnant individuals with RHD can be improved through the provision of equitable, evidence-based care, specifically through interventions targeting the causes of disparate outcomes, as presented in this scoping review. Interventions targeting RHD in pregnancy require a life-course approach, along with coordinated multisectoral and sustained government commitment by nations to reduce the global burden of RHD.

perspectives **COMPETENCY IN MEDICAL KNOWLEDGE:** There are multiple socioeconomic, cultural, and health care access-related causes of global disparities in cardiac outcomes of pregnant individuals with RHD.

**TRANSLATIONAL OUTLOOK:** This scoping review highlights targets for clinical and public health interventions to improve cardiac outcomes of pregnant individuals with RHD. It also highlights barriers to addressing such disparate outcomes, particularly the need for more qualitative data and broader representation of affected populations, which are required to understand better and ultimately address such disparities.

## Funding support and author disclosures

Dr Silversides is the Editor-in-Chief and Dr Siu is the Senior Guest Editor for cardio-obstetrics for *JACC: Advances*. Dr D’Souza holds a Canada Research Chair in Maternal Health (CRC-2021-00337). All other authors have reported that they have no relationships relevant to the contents of this paper to disclose.
