# Supplementary material for: Global Disparities in Outcomes of Pregnant Individuals With Rheumatic Heart Disease: A Scoping Review
Source: JACC Adv. 2024 Oct 30;3(12):101368. doi: 10.1016/j.jacadv.2024.101368 (PMC11734045; doi:10.1016/j.jacadv.2024.101368)
Supplement: Supplemental Tables S1-S2 [file mmc1.docx]

# **Supplemental Table 1. Sources of themes influencing outcome of pregnant individuals with Rheumatic Heart Disease**

| 1. **Preconception and antenatal considerations** | |
| --- | --- |
| 1. *Secondary antibiotic prophylaxis* | - Ongzalima CO, Greenland M, Vaughan G, Ng A, Fitz-Gerald JA, Sanfilippo FM, et al. Rheumatic heart disease in pregnancy: Profile of women admitted to a Western Australian tertiary obstetric hospital. Aust N Z J Obstet Gynaecol. 2020;60(2):302-8. |
| 1. *Late diagnosis of RHD* | - Beaton A, Okello E, Scheel A, DeWyer A, Ssembatya R, Baaka O, et al. Impact of heart disease on maternal, fetal and neonatal outcomes in a low-resource setting. Heart. 2019;105(10):755-60. - Belton S, Kruske S, Jackson Pulver L, Sherwood J, Tune K, Carapetis J, et al. Rheumatic heart disease in pregnancy: How can health services adapt to the needs of Indigenous women? A qualitative study. Aust N Z J Obstet Gynaecol. 2018;58(4):425-31. - Vaughan G, Tune K, Peek MJ, Jackson Pulver L, Remenyi B, Belton S, et al. Rheumatic heart disease in pregnancy: strategies and lessons learnt implementing a population-based study in Australia. Int Health. 2018;10(6):480-9. - Vaughan G, Dawson A, Peek M, Carapetis J, Wade V, Sullivan E. Caring for Pregnant Women with Rheumatic Heart Disease: A Qualitative Study of Health Service Provider Perspectives. Glob Heart. 2021;16(1):88. |
| 1. *Late engagement in obstetric care* | - Sullivan EA, Vaughan G, Li Z, Peek MJ, Carapetis JR, Walsh W, et al. The high prevalence and impact of rheumatic heart disease in pregnancy in First Nations populations in a high-income setting: a prospective cohort study. Br J Obstet Gynaecol. 2020;127(1):47-56. - Soma-Pillay P, Seabe J, Sliwa K. The importance of cardiovascular pathology contributing to maternal death: Confidential Enquiry into Maternal Deaths in South Africa, 2011-2013. Cardiovasc J Afr. 2016;27(2):60-5. |
| 1. **Clinical expertise and coordinated cardio-obstetrics care** | |
| 1. *Late or no cardiac consultation in pregnancy* | - Ongzalima CO, Greenland M, Vaughan G, Ng A, Fitz-Gerald JA, Sanfilippo FM, et al. Rheumatic heart disease in pregnancy: Profile of women admitted to a Western Australian tertiary obstetric hospital. Aust N Z J Obstet Gynaecol. 2020;60(2):302-8. - Sullivan EA, Vaughan G, Li Z, Peek MJ, Carapetis JR, Walsh W, et al. The high prevalence and impact of rheumatic heart disease in pregnancy in First Nations populations in a high-income setting: a prospective cohort study. Br J Obstet Gynaecol. 2020;127(1):47-56. - Soma-Pillay P, Seabe J, Sliwa K. The importance of cardiovascular pathology contributing to maternal death: Confidential Enquiry into Maternal Deaths in South Africa, 2011-2013. Cardiovasc J Afr. 2016;27(2):60-5. - Poli PA, Orang'o EO, Mwangi A, Barasa FA. Factors Related to Maternal Adverse Outcomes in Pregnant Women with Cardiac Disease in Low-resource Settings. Eur Cardiol. 2020;15:e68. |
| 1. *Lack of cardio-obstetrics clinics* | - Belton S, Kruske S, Jackson Pulver L, Sherwood J, Tune K, Carapetis J, et al. Rheumatic heart disease in pregnancy: How can health services adapt to the needs of Indigenous women? A qualitative study. Aust N Z J Obstet Gynaecol. 2018;58(4):425-31. - Vaughan G, Dawson A, Peek M, Carapetis J, Wade V, Sullivan E. Caring for Pregnant Women with Rheumatic Heart Disease: A Qualitative Study of Health Service Provider Perspectives. Glob Heart. 2021;16(1):88. - Ongzalima CO, Greenland M, Vaughan G, Ng A, Fitz-Gerald JA, Sanfilippo FM, et al. Rheumatic heart disease in pregnancy: Profile of women admitted to a Western Australian tertiary obstetric hospital. Aust N Z J Obstet Gynaecol. 2020;60(2):302-8. - Soma-Pillay P, Seabe J, Sliwa K. The importance of cardiovascular pathology contributing to maternal death: Confidential Enquiry into Maternal Deaths in South Africa, 2011-2013. Cardiovasc J Afr. 2016;27(2):60-5. - Sliwa K, Azibani F, Baard J, Osman A, Zühlke L, Lachmann A, et al. Reducing late maternal death due to cardiovascular disease - A pragmatic pilot study. Int J Cardiol. 2018;272:70-6. - Voleti S, Okello E, Murali M, Sarnacki R, Majwala A, Ssembatya R, et al. The personal and clinical impact of screen-detected maternal rheumatic heart disease in Uganda: a prospective follow up study. BMC Pregnancy Childbirth. 2020;20(1):611. - Jamal K, Raivoso M, Daniel A, Mocumbi A. Reproductive health professional's reported knowledge on diagnosis and management of rheumatic heart disease in pregnant women in Maputo, Mozambique. Int J Cardiol. 2020;317:207-10. |
| 1. *Provider knowledge and education regarding RHD assessment and management* | - Soma-Pillay P, Seabe J, Sliwa K. The importance of cardiovascular pathology contributing to maternal death: Confidential Enquiry into Maternal Deaths in South Africa, 2011-2013. Cardiovasc J Afr. 2016;27(2):60-5. - Jamal K, Raivoso M, Daniel A, Mocumbi A. Reproductive health professional's reported knowledge on diagnosis and management of rheumatic heart disease in pregnant women in Maputo, Mozambique. Int J Cardiol. 2020;317:207-10. |
| 1. **Patient health education** | |
| 1. *Patient health literacy and education* | - Diao M, Kane A, Ndiaye MB, Mbaye A, Bodian M, Dia MM, et al. Pregnancy in women with heart disease in sub-Saharan Africa. Arch Cardiovasc Dis. 2011;104(6-7):370-4. - Poli PA, Orang'o EO, Mwangi A, Barasa FA. Factors Related to Maternal Adverse Outcomes in Pregnant Women with Cardiac Disease in Low-resource Settings. Eur Cardiol. 2020;15:e68. - Schoon MG, Bam RH, Wolmarans L. Cardiac disease during pregnancy--a Free State perspective on maternal morbidity and mortality. S Afr Med J. 1997;87 Suppl 1:C19-22. |
| 1. *Patient-provider communication and rapport* | - Belton S, Kruske S, Jackson Pulver L, Sherwood J, Tune K, Carapetis J, et al. Rheumatic heart disease in pregnancy: How can health services adapt to the needs of Indigenous women? A qualitative study. Aust N Z J Obstet Gynaecol. 2018;58(4):425-31. - Vaughan G, Dawson A, Peek M, Carapetis J, Wade V, Sullivan E. Caring for Pregnant Women with Rheumatic Heart Disease: A Qualitative Study of Health Service Provider Perspectives. Glob Heart. 2021;16(1):88. - Ongzalima CO, Greenland M, Vaughan G, Ng A, Fitz-Gerald JA, Sanfilippo FM, et al. Rheumatic heart disease in pregnancy: Profile of women admitted to a Western Australian tertiary obstetric hospital. Aust N Z J Obstet Gynaecol. 2020;60(2):302-8. |
| 1. **Medical treatment during pregnancy** | |
| 1. *Cardiac medications* | - Sliwa K, Azibani F, Baard J, Osman A, Zühlke L, Lachmann A, et al. Reducing late maternal death due to cardiovascular disease - A pragmatic pilot study. Int J Cardiol. 2018;272:70-6. - van Hagen IM, Thorne SA, Taha N, Youssef G, Elnagar A, Gabriel H, et al. Pregnancy Outcomes in Women With Rheumatic Mitral Valve Disease: Results From the Registry of Pregnancy and Cardiac Disease. Circulation. 2018;137(8):806-16. |
| 1. *Anticoagulation* | - Schoon MG, Bam RH, Wolmarans L. Cardiac disease during pregnancy--a Free State perspective on maternal morbidity and mortality. S Afr Med J. 1997;87 Suppl 1:C19-22. - Zühlke L, Engel ME, Karthikeyan G, Rangarajan S, Mackie P, Cupido B, et al. Characteristics, complications, and gaps in evidence-based interventions in rheumatic heart disease: the Global Rheumatic Heart Disease Registry (the REMEDY study). Eur Heart J. 2015;36(18):1115-22a. - Elsayed AAA, Abdelaal KM, Abdelghaffar AMM, Mohamed EEH, Mahran TMA, Ahmed MSM, et al. Poor Outcome of Surgical Management of Acute Malfunctioning Mechanical Mitral Valve During Pregnancy. Should Centers with Limited Resources Find Different Options? Heart Surg Forum. 2019;22(5):E405-e10. |
| 1. *Intervention and surgery* | - Diao M, Kane A, Ndiaye MB, Mbaye A, Bodian M, Dia MM, et al. Pregnancy in women with heart disease in sub-Saharan Africa. Arch Cardiovasc Dis. 2011;104(6-7):370-4. - van Hagen IM, Thorne SA, Taha N, Youssef G, Elnagar A, Gabriel H, et al. Pregnancy Outcomes in Women With Rheumatic Mitral Valve Disease: Results From the Registry of Pregnancy and Cardiac Disease. Circulation. 2018;137(8):806-16. - Zühlke L, Engel ME, Karthikeyan G, Rangarajan S, Mackie P, Cupido B, et al. Characteristics, complications, and gaps in evidence-based interventions in rheumatic heart disease: the Global Rheumatic Heart Disease Registry (the REMEDY study). Eur Heart J. 2015;36(18):1115-22a. - Elkayam U, Bansal P, Mehra A. Catheter-Based Interventions for the Management of Valvular Heart Disease During Pregnancy. JACC: Advances. 2022;1(2):1-20. - van Hagen IM, Baart S, Fong Soe Khioe R, Sliwa-Hahnle K, Taha N, Lelonek M, et al. Influence of socioeconomic factors on pregnancy outcome in women with structural heart disease. Heart. 2018;104(9):745-52. |
| 1. **Financial resources** | |
| 1. *National level – economy* | - van Hagen IM, Thorne SA, Taha N, Youssef G, Elnagar A, Gabriel H, et al. Pregnancy Outcomes in Women With Rheumatic Mitral Valve Disease: Results From the Registry of Pregnancy and Cardiac Disease. Circulation. 2018;137(8):806-16. - Zühlke L, Engel ME, Karthikeyan G, Rangarajan S, Mackie P, Cupido B, et al. Characteristics, complications, and gaps in evidence-based interventions in rheumatic heart disease: the Global Rheumatic Heart Disease Registry (the REMEDY study). Eur Heart J. 2015;36(18):1115-22a. - van Hagen IM, Baart S, Fong Soe Khioe R, Sliwa-Hahnle K, Taha N, Lelonek M, et al. Influence of socioeconomic factors on pregnancy outcome in women with structural heart disease. Heart. 2018;104(9):745-52. |
| 1. *Patient/systems level – health insurance* | - Ongzalima CO, Greenland M, Vaughan G, Ng A, Fitz-Gerald JA, Sanfilippo FM, et al. Rheumatic heart disease in pregnancy: Profile of women admitted to a Western Australian tertiary obstetric hospital. Aust N Z J Obstet Gynaecol. 2020;60(2):302-8. - Poli PA, Orang'o EO, Mwangi A, Barasa FA. Factors Related to Maternal Adverse Outcomes in Pregnant Women with Cardiac Disease in Low-resource Settings. Eur Cardiol. 2020;15:e68. |

# **Supplemental Table 2. Details of included studies**

| **First author, year** | **Country** | **Context** | **Study type** | **Sample size** | **Relevant findings** | **Other comments and limitations noted by authors** |
| --- | --- | --- | --- | --- | --- | --- |
| Beaton, 2018 | Uganda | Hospital and health centres | Prospective longitudinal cohort study | N = 3506 screened, 58 with rheumatic heart disease (RHD) | - - - Only 3.4% of women screened with echo in pregnancy had prior diagnosis, supporting the argument to screen for RHD, as: over half required initiation of ≥1 cardiovascular (CV) medication during pregnancy; the delivery plan (delivery at a healthcare facility, caesarean section) was more likely to be modified; complication rates were high: 51% had CV complications (heart failure in 33%, pulmonary hypertension, arrhythmia, 1 death). - Population attributable risk of heart disease on maternal mortality was 88.6% in the exposed population and 10.8% in the overall population. | - Follow up was limited to 6 weeks post-partum. - Echo results were not blinded. - Need implementation and costing research to support the benefit of echo screening – postulated but not proven that screening echo reduces risk of presenting with morbidity and adverse obstetric and perinatal outcomes. |
| Belton, 2018 | Australia | Primary health care settings and hospital antenatal clinics (ANCs) | Qualitative study | N = 8 | - - Communication issues: health professionals spoke formal English with medical jargon despite women speaking Aboriginal English; none of the women were offered interpreters during observed clinical encounters; no vocabulary for ‘RHD’ or ‘heart valves’ in the Aboriginal language exists; disease descriptors were poorly explained in health education materials; health care was found to be confusing and stressful to navigate; difficulty was encountered filling prescriptions.   - Culturally-competent care was lacking: in addition to communication issues, most health practitioners held the power and controlled most health-seeking interactions. Patients consequently were disengaged and became indifferent to health practitioners. - Geographical isolation affected quality of care, especially with fragmented multi-disciplinary care (no cardio-obstetrics clinics available). Consequences included missing patient records, delayed diagnoses, and poor adherence to treatment. | - Small sample size. |
| Diao, 2011 | Senegal | Tertiary hospital | Retrospective cohort study | N = 46 pregnant women with RHD | - - Higher maternal mortality rate than other African studies of 34%, noting that percutaneous balloon mitral commissurotomy (PBMC) was not available at the study site.   - In contrast with other studies, history of cardiac event and prosthetic valve were associated with better outcomes. This was likely to be a proxy for awareness to access treatment, provider awareness of disease and management, and overall ability to access care.   - Very low educational status in most of the women, thus possibly less likely to recognise risks associated with cardiac disease and related risks of pregnancy. - Maternal death was associated with: mitral stenosis, severe tricuspid regurgitation, New York Heart Association (NYHA) functional class III/IV, and symptoms of heart failure. - Favourable outcome was associated with prior CV events, prior valve replacement, and prosthetic valves. |  |
| Elsayed, 2019 | Egypt | Tertiary hospital | Retrospective cohort study | N = 16 | - - Cohort of pregnant women requiring emergency redo mitral valve (MV) replacement due to acute malfunctioning mechanical MV who found to have poor outcomes: all presented with NYHA functional class IV, 4 of 16 died intra-/post-operatively.     - Poor compliance with anticoagulation: all patients changed their anticoagulation either without medical advice or guided by a local obstetrician (avoiding warfarin due to fear of teratogenicity) despite anticoagulation clinic protocol of warfarin due to cost of heparin and lack of anti-Xa measurement facilities. - Given the poor outcomes and poor compliance with anticoagulation, they raise the question whether mechanical valves should be avoided for women considering pregnancy in low resource settings. - The cohort was among the poorest in Egypt, are less educated and have less access to medical services. |  |
| Jamal, 2020 | Mozambique | Major referral hospitals | Descriptive observational study (surveys) | N = 73 | - - Reproductive health professionals (RHPs) from sites providing the highest level of obstetric care in Mozambique were unequipped to diagnose and manage RHD in pregnancy adequately, with: poor knowledge of clinical features and diagnostics of heart failure (satisfactory in 15% of mid-level RHPs and 44% of doctors), and management of RHD; few professionals were able to identify the drugs that are contraindicated during pregnancy (8%); most RHPs believed stand-alone scripts are the ideal way of delivering RHD medications to chronic RHD pts, only 11% doctors used chronic prescription guidelines to allow regular pharmacy monthly drug supply.   - Issues with service organisation and access to guidelines: 11% of doctors and 54% of mid-level RHPs have arrangements to evaluate women with suspected or confirmed cardiac disease; 31% of all RHPs have access to acute rheumatic fever/RHD guidelines or protocols for management in pregnancy; 74% had no access to contraceptive-specific guidance for women with RHD. | - Limitations of survey-based self-administered study: bias or issues with accuracy of responses, generalisability. |
| Ongzalima, 2020 | Australia | Tertiary centre | Retrospective cohort study | N = 54 | - Low rates of antibiotic secondary prophylaxis: 23.1% of non-Indigenous and 68.3% of Indigenous women with RHD.   - Lack of understanding and trust for Aboriginal women resulted in service disengagement and non-attendance to antenatal clinics, increasing risk of missed RHD diagnosis or severity.   - Increased health workforce awareness of guidelines for use of prophylaxis in management of RHD is required. - Antenatal care attendance was suboptimal in 40.4% of women, with significantly lower attendance among Indigenous versus non-Indigenous women (p= 0.0078). - Social challenges to care include: distance to maternity service, lack of culturally-safe antenatal care, and fear of not birthing near family.   - During admission, 65.9% of Indigenous patients had contact with an Aboriginal liaison officer. - Proportion of women with severe RHD: 40.7%. - There was limited direct access to cardiology support and intensive care at the study site. | - Small sample size, retrospective nature of study. |
| Poli, 2020 | Kenya | Tertiary hospital | Longitudinal cohort study | N = 91 | - - Factors associated with adverse maternal outcomes (cardiac and obstetric) for women with RHD were: limited access to quality antenatal care, low maternal education level, home births, level of facility that provided care; lack of health insurance contributed to poor outcomes due to limited access to ICUs in private hospitals when public hospital ICUs were full. - Rate of cardiac adverse events was higher than in other studies. - Over half of women who were diagnosed with cardiac disease during pregnancy were not receiving treatment. - Surgical intervention pre-pregnancy was not predictive of better outcomes (in contrast to CARPREG II scoring), likely due to low numbers (only available in 5 sites in Kenya, with many patients unable to afford it). This highlights the issue of generalisability of scoring systems and guidelines to low resource settings. |  |
| Schoon, 1997 | South Africa | Tertiary hospital | Retrospective cohort study | N = 164 pregnant women with RHD | - Ignorance about pregnancy complications or therapy was evident.   - Late booking and poor understanding of the implications of a pregnancy in a patient with cardiac disease were major contributors to morbidity and mortality.   - An important risk factor for death was inability to comply with anticoagulation: with valve thrombosis and death with non-compliance with both warfarin and heparin, and severe hemorrhage also with both anticoagulants. |  |
| Sliwa, 2018 | South Africa | Tertiary hospital | Prospective pilot study | N = 269 pregnant women with cardiac disease | - Targeted interventions are associated with significant reduction in peripartum heart failure admissions and mortality: Cardiac-obstetric care, continuation of beta-blockers while pregnant and early start of heart failure medication postpartum when indicated (angiotensin inhibitor and aldosterone antagonists). |  |
| Soma-Pillay, 2016 | South Africa | National database: Confidential enquiry into maternal deaths | Retrospective cohort study | N = 169 | - Peripartum cardiomyopathy (34%) and complications of RHD (25.3%) were the major causes of maternal death in 2011-2013. - Avoidable factors contributing to death included: delay in patients seeking help (41.5%), lack of expertise of medical staff managing the case (29.7%), delay in referral to the appropriate level of care (26.3%) and inappropriate action (36.4%). - Most patients booked late, and only 33.7% had a specialist as an antenatal care provider. - Discussed the need for echo screening, early diagnosis and referral, and cardio-obstetric clinic management – but does not provide evidence that lack of these contribute to the deaths. | - Data (e.g. factors contributing to death) were not specific to RHD patients. - Mortality was only captured within 42 days post-partum. - Diagnosis was not confirmed for many cases e.g. made by a junior doctor and not verified, or the patient died prior to reaching higher level hospital. |
| Sullivan, 2020 | Australia and New Zealand (Aus/NZ) | Hospital-based maternity units | Prospective cohort study | N = 311 | - Pregnant women with RHD in Aus/NZ are more likely to be Indigenous: 78% of Aus, 90% of NZ cohorts.   - Rate of RHD amongst pregnant Indigenous Australians was 2.22%, versus 2.5% in all Indigenous Australians (no lower than in the non-pregnant population), 50-fold higher than the national prevalence, and on par with low-income settings. - Late diagnosis of RHD in pregnancy was common and associated with adverse outcomes (NYHA class deterioration, maternal admission to intensive and coronary care units (ICU/CCU), general anaesthesia requirement for caesarean section).   - Antenatal care received by women in the study was suboptimal: only received by 30% in the first trimester, versus 60% of all Australian women.   - Cardiac care was suboptimal: 33% received no cardiac care and 15% did not have an echo during pregnancy. - Rates of adverse outcomes were low: 1 woman died, 10% admitted to CCU/ICU postpartum. | - Data was incomplete as women accessed care at different sites. - Indigenous status may have been under-reported. |
| Van Hagen, 2018 (1) | Global | Tertiary hospitals | Prospective registry (ROPAC) | N = 2,924 (89 centres, 30 countries) | - Analysis of ROPAC (registry of pregnant women with cardiac disease) specifically to identify the contribution of socioeconomic factors (at the country-level) upon the variance in maternal outcomes.   - Differences between centres and countries were identified: women in medium/high versus very high human development index (HDI) countries were more likely to have heart failure, modified World Health Organisation (mWHO) class III/IV; and less likely to have had cardiac intervention. - Gini coefficient and country-specific birth rate explained 4% of the variance in outcomes (maternal cardiac event). However patient characteristics (maternal age, NYHA classification and mWHO risk classification) were stronger predictive factors of outcome and explained 37% of the variance. | - Socioeconomic range and representation was limited: the study included countries with medium to very high HDI (75% from emerging countries were from Egypt), centres were university/tertiary referral centres, and individual sociodemographic data was not collected (only collected at the country level). - Follow up data only included 1 week post-partum. |
| Van Hagen, 2018 (2) | Global | Tertiary hospitals | Prospective registry (ROPAC) | N = 2,924 (89 centres, 30 countries) | - Only 40% of women with mitral stenosis (MS) and heart failure were treated with beta blockers, highlighting the gap between guideline recommendations and clinical implementation, and the need for wider education. - Emerging versus advanced economy countries had more women with severe MS but a lower number of valvular interventions. - Differences in outcomes between emerging versus advanced economies: found for birth weight and pregnancy duration (advanced economies had higher induction and Caesarean section rates), but there was no difference in maternal outcomes (albeit with sample sizes). - Half of the women with severe MS required hospital admission for heart failure during pregnancy, with speculation that pre-pregnancy or antenatal PBMC may prevent this (performed in 16 of 273 – 5.8% patients with MS during pregnancy. | - Socioeconomic range and representation was limited: included countries had medium to very high HDI (75% from emerging countries were from Egypt), included centres were university/tertiary referral centres, and individual sociodemographic data was not collected (only collected at the country level). - Follow up data only included 1 week post-partum. |
| Vaughan, 2018 | Australia | Maternity units in hospitals and community clinics | Population-based study | N = 192 | - - Challenges to effective provision of care was identified across several tiers, relating to: case ascertainment (variable amount and quality of information provided in echo report used to identify cases, gaps in accurate identification of cases and also false-positives using ICD10 coding); burden of reporting (echo reports were often not included in patient notes); health information systems (lack of cohesion in standard data collecting systems with multiple systems/ sources e.g. mix of electronic and paper-based records, multiple data systems in primary and remote networks that do not communicate, and ineffective transfer of information between and across health services and jurisdictions). | - Miscarriages and terminations were not captured in units participating in the surveillance system. - Limited applicability to LICs. - Under-reporting of Indigenous status. |
| Vaughan, 2021 | Australia | Hospitals and community-based centres | Qualitative study | N = 19 | - Lack of integrated women-centred health care during pregnancy for women with RHD from the perspective of health professionals.   - Health system challenges contributing to the above included insufficient: workforce, access to services, clinical and cultural awareness; and fragmented provision of health services (from siloed processes, knowledge and clinical care).   - Consequences included: referrals (e.g. for echocardiography) not being actioned, and test results not being received. - Pronounced issues/consequences of the above points in remote Australia, where RHD prevalence is highest. - Lack of effort to increase health literacy (specifically regarding RHD) in a culturally/ linguistically appropriate manner. - *“Emerging from the study findings was a recognition that optimal care requires a woman-centered life course approach that supports transition to adult care, considers surgery and other interventions in the context of reproductive health and preconception care, as well as pregnancy and postpartum care.”* | - Small and heterogenous study sample. - Limited transferability of study findings (as this study was specific to Australian Indigenous women). |
| Zuhlke, 2015 | Global registry (REMEDY) | Hospitals | Prospective RHD registry | N = 3343 patients with RHD, including 73 pregnant women | - - Anticoagulation with warfarin was prescribed in 69.5% of patients, and 20.6% of pregnant women. 28.3% of those prescribed anticoagulation had a therapeutic INR. - Contraception was only used by 3.6% of 1825 women of child-bearing age. - Valvuloplasty and valve surgery use was lower in low- versus upper-middle income countries, despite higher prevalence of patients with RHD and left ventricular dysfunction requiring these interventions in low-income countries. |  |
